# Invasive aspergillosis in solid organ transplant patients: diagnosis, prophylaxis, treatment, and assessment of response

**DOI:** 10.1186/s12879-021-05958-3

**Published:** 2021-03-24

**Authors:** Dionysios Neofytos, Carolina Garcia-Vidal, Frédéric Lamoth, Christoph Lichtenstern, Alessandro Perrella, Jörg Janne Vehreschild

**Affiliations:** 1 Service des Maladies Infectieuses, Hôpitaux Universitaires de Genève, Rue Gabrielle-Perret-Gentil 4, Geneva, Switzerland; 2 Servicio de Enfermedades Infecciosas, Hospital Clínic de Barcelona-IDIBAPS, Universitat de Barcelona, FungiCLINIC Research group (AGAUR), Barcelona, Spain; 3 Infectious Diseases Service, Department of Medicine, Lausanne University Hospital, 1011 Lausanne, Switzerland; 4 Department of Laboratories, Institute of Microbiology, Lausanne University Hospital, Lausanne, Switzerland; 5 Department of Anaesthesiology, Heidelberg University Hospital, Im Neuenheimer Feld 110, Heidelberg, Germany; 6 VII Department of Infectious Disease and Immunology, Hospital D. Cotugno, Naples, Italy; 7 CLSE-Liver Transplant Unit, Hospital A. Cardarelli, Naples, Italy; 8 Medical Department II, Hematology and Oncology, University Hospital of Frankfurt, Frankfurt, Germany; 9 Department I for Internal Medicine, University Hospital of Cologne, Cologne, Germany; 10 German Centre for Infection Research, partner site Bonn-Cologne, University of Cologne, Cologne, Germany

**Keywords:** *Aspergillus*, Invasive pulmonary aspergillosis, Microbiome, *Mucorales*, Mucormycosis, Solid organ transplantation

## Abstract

**Background:**

Invasive aspergillosis (IA) is a rare complication in solid organ transplant (SOT) recipients. Although IA has significant implications on graft and patient survival, data on diagnosis and management of this infection in SOT recipients are still limited.

**Methods:**

Discussion of current practices and limitations in the diagnosis, prophylaxis, and treatment of IA and proposal of means of assessing treatment response in SOT recipients.

**Results:**

Liver, lung, heart or kidney transplant recipients have common as well as different risk factors to the development of IA, thus each category needs a separate evaluation. Diagnosis of IA in SOT recipients requires a high degree of awareness, because established diagnostic tools may not provide the same sensitivity and specificity observed in the neutropenic population. IA treatment relies primarily on mold-active triazoles, but potential interactions with immunosuppressants and other concomitant therapies need special attention.

**Conclusions:**

Criteria to assess response have not been sufficiently evaluated in the SOT population and CT lesion dynamics, and serologic markers may be influenced by the underlying disease and type and severity of immunosuppression. There is a need for well-orchestrated efforts to study IA diagnosis and management in SOT recipients and to develop comprehensive guidelines for this population.

## Background

Invasive mold infections (IMI), in particular invasive aspergillosis (IA), are a relatively rare complication in solid organ transplant (SOT) recipients [1–3], albeit associated with high rates of graft loss and mortality [4]. The overall incidence of IA among SOT recipients remains below 10% and varies depending on the organ transplanted [1, 5]. IA post-SOT is associated with high overall mortality, with 3-month rates as high as 15–25% in non-liver and up to 80–90% in liver SOT recipients (Table 1) [1, 2].

**Table 1:** Epidemiology of invasive aspergillosis in SOT recipients. The large variations of the overall mortality rates in heart and kidney recipients can be explained by the corresponding variations in follow-up in the different studies (3-months [1, 3, 5] or 12-months [6, 7])

Population	Incidence (%)	Overall mortality (%)	References
Heart	3.5–26.7	36–66.7	[1, 3, 5, 8, 9]
Kidney	1.2–4	4–25	[1, 3, 5]
Liver	1–4.7	83–88	[1, 3, 5]
Lung	8.3–23.3	4.2	[1, 3, 5]

Considering the devastating consequences of IA in SOT recipients [3, 5], mold-active primary prophylaxis is used routinely in some transplant centers [10]. However, the administration of broad-spectrum antifungal prophylaxis in the SOT setting remains controversial, considering the lack of available evidence, significant drug-drug interactions (particularly between azoles and some immunosuppressive agents), costs, selection for resistant pathogens (in particular, *Candida* spp.) and the risk of breakthrough IMI caused by resistant molds [11]. Attempts to stratify antifungal prophylaxis based on identification of IA predictors have largely failed [12]. In addition, although the pathophysiology of IA and the effects of the intensity and duration of immunosuppressive therapy on IA are now better appreciated [5], a large array of additional risk factors appear to be of variable importance for different transplanted organs (Table 2).

**Table 2:** Risk factors for invasive aspergillosis in SOT recipients. Herbrecht et al. [13] have listed the general risk factors for invasive fungal infections in haemato-oncological patients and solid organ transplant recipients, but the list is continuously increasing, and presently includes a number of additional factors [14–16], among them influenza, [17, 18]

	Risk factors	References
Heart	Reoperation; CMV infection; post-transplantation hemodialysis; presence of another patient with IA in the transplant program 2 months before or after the procedure; rejection, admission to the ICU, mechanical ventilation, and extracorporeal membrane oxygenation (ECMO)	[19, 20]
Kidney	Bloodstream infections; pre-transplant chronic pulmonary obstructive disease; impaired graft function; long-term dialysis prior to transplantation; serious post-transplant infections	[21, 22]
Liver	MELD score, choledochojejunostomy; anastomosis; bacterial infections in the first month and absence of antifungal prophylaxis; cytomegalovirus (CMV) reactivation; renal failure; hemodialysis; re-transplantation or transplantation for fulminant hepatic failure; reoperation	[5, 12, 22–24]
Lung	Single lung transplantation; pre- and post-transplant colonization with *Aspergillus* spp., early airway ischemia, CMV infection, rejection	[25, 26]

Here we briefly discuss current practices and limitations in the diagnosis, prophylaxis, and treatment of IA, as well as means of assessing treatment response in SOT recipients.

## Methods

**Fig. 1:**
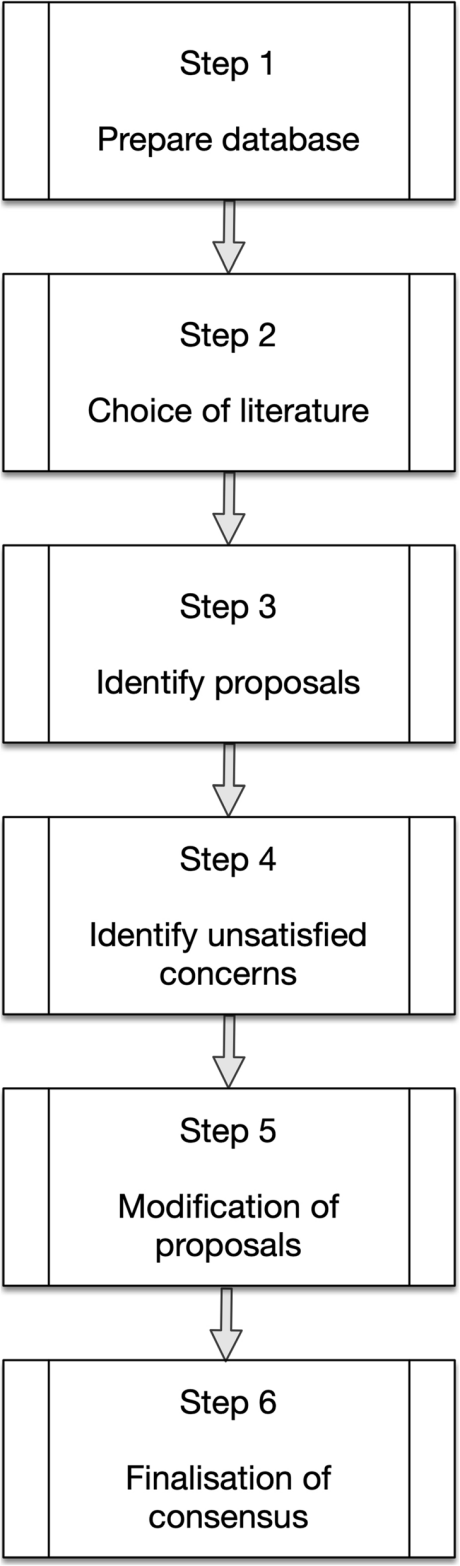
Basic steps to consensus decision making used in this work

This consensus document was the product of an expert panel based on a consensus, decision-making process to produce an unbiased, independent and high-quality manuscript. Participants were chosen on the basis of their expertise in the field of medical mycology and transplantation medicine. Each expert was assigned to one of the following topics: epidemiology, diagnosis, radiological and clinical presentation, treatment and clinical outcomes of IA in SOT recipients. Literature review was performed through the PubMed database for articles written in English between 2000 and 2018 on IA-epidemiology, IA-diagnosis, IA-treatment, and IA-clinical outcomes in SOT. All participants reviewed individually the available literature on the topic they were assigned to and chose the most relevant data to present and discuss. Critical discussion of all data was subsequently performed by all experts and consensus decisions were made on each topic. Agreement by all members of the panel was required for a recommendation to be made and included in the consensus document. Each author provided a draft manuscript on their assigned topic. The final manuscript was reviewed and accepted as such by all authors. The organization plan used is provided in Fig. 1.

## Results

### Diagnostic workup

The diagnosis of IA relies on a multitiered approach that should consider risk factors and the local epidemiology, as well as the performance and limitations of the available diagnostic tools [14, 15]. IA diagnosis warrants a comprehensive and rigorous workup, including a combination of histopathology, microbiology, serology, and imaging data in the relevant clinical setting. However, these considerations are predominately based on data generated from patients with hematologic malignancies and hematopoietic cell transplantation (HCT) recipients. Based on the limited data available on diagnostic tools in SOT recipients, imaging and biomarkers, such as the galactomannan enzyme immunoassay (GM EIA), appear to perform less optimally compared to neutropenic patients [1, 2, 4, 27]. Lack of prospective high-quality clinical studies on the performance of imaging, microbiology, and/or laboratory biomarkers for the diagnosis of IA in SOT recipients significantly limits our ability to establish a definitive diagnosis of IA in SOT setting and requires additional efforts to optimize the use of these tools. The work done by the International Society for Heart and Lung Transplantation (ISHLT) for lung and heart transplant recipients [28] should be expanded to all other forms of transplantation.

### Microbiology

Traditional diagnostic approaches include staining with Gomori’s methenamine silver or periodic acid–Schiff (PAS) stains and fungal cultures of clinical specimens, with a historical sensitivity that varies between 20 and 70% [15, 29–35]. Sensitivity and positive predictive values heavily depend on the quality of the specimen obtained (sputum versus bronchoalveolar lavage, BAL), severity of disease, and organism inoculum [15, 34, 36–38].

To fill in the gap, additional non-culture methods have been introduced in clinical practice, including fungal biomarkers, such as the GM EIA and beta-D-glucan (BDG), and molecular testing with polymerase chain reaction (PCR). Among the available biomarkers and despite its established role in hematologic patients [39], the GM EIA performs rather poorly in serum samples of SOT recipients, with a sensitivity ranging from 30 to 58% [1, 27, 40–42]. In contrast, the performance of GM EIA in BAL of SOT recipients appears to be better, with a sensitivity in the range of 67–100% [5]. The BDG test remains of relatively poor value, due to its lack of specificity for *Aspergillus* spp. [43–49] and limited sensitivity particularly in liver transplant recipients, ranging between 58 and 65% [42, 43, 50]. Although currently not widely used, *Aspergillus* specific PCR in the serum or BAL has been shown to offer additional diagnostic value in both neutropenic and non-neutropenic patients [51–53]. However, the validation of new diagnostic tests for the diagnosis of IA in SOT recipients remains problematic, due to the lack of easily applicable gold standards and the constantly decreasing rates of biopsy performance in clinical practice.

Notably, the worldwide shift towards establishing a diagnosis of IA using fungal biomarkers and without isolating the pathogen may pose significant problems in the management of IA in the near future. Fungal pathogen availability is important for antifungal susceptibility testing, a crucial step to optimize the management of patients with IA. This is even more pertinent now, as there have been increasing reports of worldwide emergence of multi-triazole resistance among *Aspergillus fumigatus*, in most cases due to a TR34/L98H mutation [54–56], and intrinsically resistant, cryptic species in the *Aspergillus fumigatus* complex, such as *A. lentulus*, *A. udagawae*, and *A. viridinutans*, are emerging as important pathogens in this universal antifungal prophylaxis era [57, 58]. *A. calidoustus* exhibits some levels of intrinsic azole resistance and has also been reported as an emerging cause of IA in non-neutropenic transplant patients receiving azole prophylaxis [11, 59], whereas *A. terreus* is intrinsically resistant to amphotericin B [60]. Several molecular protocols are available to reliably identify and characterise azole-resistant *Aspergillus* isolates [61–63], including at least a commercial kit [63, 64]. Recently a specific TaqMan Real-Time PCR has been shown to detect triazole-resistant strains of *A. fumigatus* even in the presence of only a low percentage of resistant cells [65]. In any case, antifungal susceptibility testing (AST), either by conventional in vitro AST [66, 67], or new methods such as MALDI-TOF mass spectrometry assays [68], is of critical value for the treatment of such patients and should be performed regularly in patients with IMI when a pathogen is identified, at least in regions with known resistance problems, as recommended also by the ESCMID-ECMM-ERS guideline [15].

#### Other diagnostic laboratory tools

The lateral-flow device (LFD) [69] applied to BAL has been shown to provide a reliable diagnosis of IA [70]. The technique has a short turnaround time, is easy to use and cost effective [71]. When combined with a quantitative PCR, it has contributed to detect invasive pulmonary aspergillosis in immunocompromised patients [72] and its performance was superior to that of the GM EIA test in SOT recipients [73]. The LFD, however, appears to have a reduced sensitivity in the presence of antifungal treatment [74], and, contrary to the GM test, provides only qualitative data [75]. In addition, to our knowledge, only one validated commercial kit is available [71].

#### Inflammatory markers

Non-specific inflammatory markers, such as the C-reactive protein (CRP) or fibrinogen, and pro-inflammatory markers, such as cytokines and procalcitonin (PCT), have so far not been evaluated in SOT recipients for the diagnosis of IA. In neutropenic patients with IA, high initial interleukin (IL)-8 and continuously elevated IL-6, IL-8, and CRP levels during treatment have been shown to be early predictors of therapeutic failure, suggesting that cytokine and CRP profiles could be helpful to identify non-responders, guiding to targeted, early changes in antifungal treatment [76]. The assessment of other host biomarkers, such as haptoglobin (Hp) and annexin A1 [77] or cytokines such as serum IL-10, and interferon-γ [76], needs further evaluation [78, 79]. None of these markers, however, are sufficiently specific to play a role in the IA diagnosis in this population.

### Imaging

Computerised tomography (CT) is an important tool to diagnose IA [14, 15]. The appearance of CT findings in SOT recipients with IA may not always be similar to that observed in neutropenic patients, as the classic halo sign, air-crescent sign and well-defined nodular lesions seem to be less frequent [80, 81]. Conversely, non-specific radiologic manifestations, including consolidations, pleural effusions, and ground-glass opacities, have often been described in SOT recipients with invasive pulmonary aspergillosis [82]. Some of these findings were not included in the revised European Organisation for Research and Treatment of Cancer – Mycoses Study Group (EORTC-MSG) definition consensus guidelines for the diagnosis of IA [83] and have now been partly included in the updated criteria for the diagnosis of invasive fungal infections (IFI) by the EORTC-MSG group [84]; they have been used, however, in modified diagnostic criteria among SOT recipients in observational retrospective studies [1, 85]. Lack of validation of these findings may pose additional problems in the conduction of clinical trials, particularly with regards to the accuracy and homogeneity of IA diagnosis in these settings. However, the existing body of literature and accumulating clinical experience call for collaborative action to improve our understanding of CT findings indicative of IA in SOT recipients. In the meantime, suspicious pulmonary lesions of unknown origin should prompt a rigorous workup, including bronchoscopy and/or biopsy. Recent research has suggested the potential utility of CT pulmonary angiography, using vessel occlusion signs as specific indicator of IA in hematology patients [86–88], but this strategy has not yet been evaluated in the SOT population.

### Treatment

Limited treatment options for SOT recipients with IA are available (Table 3). This is, in part, due to the complicated profile of SOT recipients, who comprise a wide array of underlying pathologies (from kidney to liver to lung transplant recipients) and underlying organ dysfunctions. Furthermore, in SOT recipients, specific pharmacokinetic/pharmacodynamic (PK/PD) and drug-drug interaction considerations significantly impact antifungal treatment options [91–94]. The distinct toxicity profiles of different antifungal drugs adds to therapeutic complexity in the setting [95]. Potential liver toxicity associated with triazoles may create additional problems in liver transplant recipients [96], whereas the nephrotoxicity associated with conventional but also with the lipid formulations of amphotericin B limits the utility of these agents in kidney transplant recipients and patients with pre-existing renal failure due to other reasons, including administration of calcineurin inhibitors [97]. Co-administration of triazoles, particularly voriconazole and posaconazole, with the most common immunosuppressive agents in SOT recipients, such as tacrolimus and sirolimus, is another major concern because of potential important drug-drug interactions [98].

**Table 3:** Primary antifungal treatment options for the treatment of IA and special considerations in solid organ transplant recipients

Agent	Dose	Recommendation	Potential Adverse Events	Potential Drug Interactions	Additional Considerations	Monitoring
Voriconazole	Induction: 6 mg/kg IV^a^ every 12 h the first day Maintenance: 4 mg/kg IV^a^, 200-300 mg PO twice daily	1st line [14]	-Hepatotoxicity^b^ -Visual changes -Neurologic toxicity -Rash and photosensitivity -Periostitis -QTc prolongation^c^	-Sirolimus^d^ -Tacrolimus^d^ -Cyclosporine^d^	-Non-linear pharmacokinetics -Strong inhibitor of CYP3A4 -Moderate inhibitor of CYP2C19 and 2C9 -Metabolized via CYP2C19, 2C9 and 3A4 - < 2% of voriconazole is excreted in the urine	-Liver function tests −12-lead ECG^f^ -Voriconazole TDM^d^ -Sirolimus, tacrolimus, and cyclosporine TDM^d^
Isavuconazole	Induction: 200 mg three times daily the first 2 days Maintenance: 200 mg daily	1st line [15] Primary alternative [14]	-Hepatotoxicity^b^	-Sirolimus^e^ -Tacrolimus^e^ -Cyclosporine^e^	-Linear pharmacokinetics -Moderate inhibitor of CYP3A4 -Metabolized via CYP3A4 -Isavuconazole may cause QTc shortening	-Liver function tests -Sirolimus, tacrolimus, and cyclosporine TDM^e^
Liposomal Amphotericin B	3–5 mg/kg daily IV	Primary alternative [14, 15]	- Nephrotoxicity^g^			-Renal function and electrolytes

*IV* Intravenous, *PO* Oral, *ECG* Electrocardiogram, *TDM* Therapeutic Drug Monitoring

^a^IV voriconazole is not recommended in patients with renal dysfunction (glomerular filtration rate < 50 mL/min) due to the potential of nephrotoxicity associated with the IV formulation vehicle of cyclodextrin

^b^Hepatotoxicity was significantly less frequent in patients treated with isavuconazole as compared to voriconazole in a prospective randomized clinical trial [89]

^c^Isavuconazole is associated with shortening of the QTc interval

^d^Voriconazole may significantly increase sirolimus levels, therefore close monitoring of sirolimus TDM is recommended in case of co-administration. Significant dose reductions of sirolimus, tacrolimus and cyclosporine are commonly required when any of these agents is co-administered with voriconazole

^e^Early data suggest that isavuconazole administration does not significantly affect blood concentrations of sirolimus and tacrolimus [90]. Until more data become available, it is advised to closely monitor immunosuppression TDM while co-administered with isavuconazole

^f^In cases of baseline QTc prolongation and/or co-administration with QTc prolonging agents, such as macrolides and fluroquinolones, regular QTc monitoring is recommended

^g^Additional, noteworthy toxicities include hypomagnesaemia, renal tubular acidosis, and elevated liver function tests

Voriconazole is the first-line option of IA therapy based on international guidelines [14, 25]. The use of voriconazole in SOT recipients, however, may be hindered due to potential drug-drug interactions, predominately with concomitantly administered immunosuppressive agents, including cyclosporine, tacrolimus and sirolimus [99–103]. Rigorous therapeutic drug monitoring (TDM) of these drugs is warranted to avoid potentially severe toxicities from overdosing [14, 15, 104, 105]. In addition, the use of voriconazole TDM [106, 107] is recommended to avoid off-target trough serum levels [108] and to identify treatment failure or toxicity because of inadequate drug exposure [15, 107, 109]. Despite the utility and significant benefits attained with regular TDM, voriconazole is frequently avoided in clinical practice, due to its adverse event profile, including hepatotoxicity and neurological and psychiatric symptoms, particularly early post-SOT and in liver transplant recipients.

Isavuconazole has demonstrated equal efficacy compared to voriconazole in patients with hematologic malignancies and HCT recipients [89], and is currently recommended for the treatment of IA by national and international guidelines [14, 15]. Notably, isavuconazole has shown lower rates of liver and neurological toxicities and has fewer drug-drug interactions, including with tacrolimus and sirolimus [90]. Considering its water soluble profile, the intravenous (IV) formulation of isavuconazole does not require co-administration with cyclodextrin [110], therefore – and unlike IV voriconazole – it may be considered even in patients with borderline renal dysfunction. Despite the currently very limited relevant data, isavuconazole represents a potentially useful agent for the treatment of IA in SOT recipients, particularly early after a liver and/or kidney transplant, to avoid significant drug interactions and associated toxicities. Although not currently recommended, isavuconazole TDM may potentially inform clinical practice, considering limited data suggesting moderate interpatient variability and concentrations affected by patients’ gender and weight and hemodialysis requirements [111, 112].

Liposomal amphotericin B (L-AmB) monotherapy is considered second line treatment for patients with IA, but it may be used in cases when triazole administration is contraindicated due to potential drug interactions and hepatotoxicity or in the presence of azole-resistant *Aspergilli* [14, 15]. The role of echinocandins in the treatment of IA in SOT recipients is not clear, and the recent ESCMID-ECMM-ERS guidelines consider the use of echinocandins only as primary prophylaxis and as combination treatment for infections due to azole-resistant *Aspergilli* [15]. In addition, there are no convincing data that combining a broad-spectrum azole or a lipid-formulation of amphotericin B with an echinocandin is beneficial in the management of IA in SOT recipients, although combination antifungal therapy is prescribed in up to one third of SOT recipients with IA [113, 114]. Combination treatment with anidulafungin and voriconazole was not associated with higher survival in high-risk hematological patients and allogeneic hematopoietic cell transplant recipients when compared with monotherapy with voriconazole, although a potential benefit was noted in post-hoc analyses of patients with a positive GM EIA optical density between 0.5 and 1.5 [115]. Based on the existing available data, combination therapy is not recommended for the treatment of IA in SOT recipients.

Posaconazole and itraconazole are included in the 2016 update of the diagnosis and management of aspergillosis by the IDSA [14, 15] but their use is limited to alternative salvage therapy. New drugs currently under development such as F901318 (olorofim) [116, 117] or ibrexafungerp (currently undergoing Phase II and III clinical trials) [118] may play a role in the management of IA, but more data are needed before further conclusions can be drawn.

###  Antifungal prophylaxis

Antifungal prophylaxis with posaconazole is recommended in high-risk hematological patients with profound neutropenia or with high-grade graft-versus-host-disease [14, 119], but primary antifungal prophylaxis is not routinely recommended for all SOT recipients. Furthermore, antifungal prophylactic strategies may vary significantly across different transplant centers [120–122]. In fact, based on the low incidence of IA and other IMI in the SOT population, variable timing of their occurrence post SOT, and lack of robust data to show efficacy in preventing IMI, most centers do not apply universal antifungal prophylaxis in SOT recipients. A recent prospective randomized clinical trial for antifungal prophylaxis of liver transplant recipients based on prior identified risk factors for IA failed to show significant benefit, at least partly due to the low number of patients diagnosed with IA [12]. The use of areosolized amphothericin B lipid complex as a standard mold-active prophylaxis appeared to be beneficial when used for up to 18 days after surgery [123]. A preemptive therapy is currently recommended only in lung transplant recipients, while a targeted prophylaxis is favored in liver and heart transplant recipients [25]. Overall, most existing data on prophylaxis and preemptive therapy of IA are based on retrospective cohort and case-control studies.

### Breakthrough fungal infections

Breakthrough IMI with reduced susceptibility to the available antifungal agents is also a major concern that should be considered in the decision-making process of universal antifungal prophylaxis. Breakthrough IMI under mold-active prophylaxis may be caused by molds that have been selected due to intrinsic or acquired resistance to the prophylactic agent used, the latter in case of suboptimal absorption and/or tissue concentration of the administered antifungal prophylaxis [11, 124, 125]. There are few scattered data on the incidence of breakthrough IMI, with the vast majority of studies reporting, almost exclusively, on hematology patients [119, 124–130]. Recently, data on breakthrough IFI in lung transplant recipients undergoing mold-active prophylaxis were reported [123]. Based on the very limited information on SOT recipients, breakthrough IMI causes vary across studies and seem to be dependent on the local epidemiological landscape and other variables. *Mucorales*, rare multi-drug resistant *Aspergillus* spp. and a shift towards rare mold species with intrinsic azole resistance compared to non-breakthrough IMI have been described as the major cause of breakthrough IMI [11, 124, 126]. Among *Aspergillus* spp., *A. calidoustus* was found to be the predominant cause of breakthrough IMI in a recent study including also SOT recipients [11]. Considering the high mortality and treatment failure rates of breakthrough IMI and the limited available therapeutic options, recent guidelines and expert reports recommend an individualized approach that considers a careful evaluation of the local epidemiology, clinical vigilance, and careful management, including empirical change of antifungal drug class [14].

### Monitoring response

Until now, lack of reliable and objective follow-up markers has made clinical response assessment, even in the setting of clinical trials, a complex and cumbersome task. Consensus criteria to assess clinical response to antifungal therapy in clinical trials of IMI have already been published [131]. The most recent ESCMID-ECMM-ERS guidelines on the diagnosis and management of IA propose to use a composite outcome of clinical, radiological and mycological criteria to assess IA treatment response [15]. These guidelines, however, have not been validated, are largely centered on hematological patients, and were created predominantly for use in clinical trials.

This is of relevance when considering treatment duration and response in SOT recipients with IA. Most SOT patients require life-long immunosuppression. Importantly, the level of immunosuppression, an important intervention to achieve faster and more complete control of IA, cannot be substantially reduced in most cases. These considerations may represent valid arguments for more prolonged treatment courses or secondary prophylaxis in SOT recipients. Because clinical signs of IA are scarce and non-specific, patient follow-up is primarily based on monitoring the radiological response and decline of serum GM EIA. Radiological response in hematological patients is notoriously delayed with an initial increase in the size of lung lesions during the first weeks despite appropriate antifungal therapy [132–136]. Data on IFI-related changes in lesion size and shape in SOT patients, in whom radiological lesions are often of different nature compared to the classical nodular lesions of hematological patients, are sparse. Until more studies provide additional insights on the evolution of radiological lesions in SOT recipients treated for IA, development of new lesions and continued increase in lesion size under treatment should alert the treating physicians for potential treatment failure and additional actions to be taken. Close follow-up of imaging findings should be performed until other clear indicators of improvement can be observed. Notably, definitive recommendations on the type and timing of clinical response in SOT recipients with IA are not currently available.

Serum GM EIA level kinetics have been reported to be good predictors of outcome of IA in hematological patients [6, 45, 75, 91, 137, 138], but their value in SOT recipients needs to be verified. In addition, only approximately one third of IA cases in SOT patients have positive GM EIA testing [1]. High negative predictive values of successful response to IA therapy have been reported when several biomarkers (GM EIA, panfungal quantitative PCR, and CT imaging) were combined [7]. The IDSA and ESCMID-ECMM-ERS guidelines [14, 15], however, do not provide specific advice as to which parameters and in which combination they should be used. Non-specific markers, such as CRP and cytokines have been proposed to predict treatment outcome in hematological patients [79], CRP has been described as a good independent prognostic factor in patients with acute invasive fungal rhinosinusitis [139], and its levels remained persistently elevated in non-responder leukemia patients [78]. CRP, however, is not specific for fungal infections and as SOT recipients are particularly prone to concomitant or intercurrent bacterial infections, the specificity of assessment of response to IA therapy in SOT patients based on CRP and other non-specific inflammatory markers needs to be verified in well-designed clinical trials.

## Conclusions

IA is a rare but often deadly disease in SOT recipients. Prevalence varies across transplanted organs, with lung and liver transplant patients being more often affected. Compared to neutropenic patients, diagnosis of IA in SOT recipients requires a high degree of clinical suspicion and awareness, especially because established diagnostic tools, such as the GM EIA test and CT, do not provide the same sensitivity and specificity observed in the neutropenic population.

As in other populations, IA treatment relies primarily on mold-active triazoles, but potential interactions with immunosuppressants and other concomitant therapies need special attention. High-dose corticosteroid treatment [140] and other immunosuppressive agents have been identified as significant risk factors for IA in high-risk patient categories [5, 17, 141–147]. Reduction of immunosuppression, if possible, should be considered as a part of the treatment strategy. Assessing response is already challenging in the settings of hematological and oncological patients, where clinicians typically rely on reduction of lesion size or emergence of air-crescent signs and cavities in CT scans as well as of serology (GM EIA, BDG) and inflammatory (fever and CRP) biomarkers. These criteria, however, have not been sufficiently evaluated in the SOT population and CT lesion dynamics as well as serologic markers are known to be influenced by the underlying disease as well as type and severity of immunosuppression. One major difference of SOT recipients compared to other patient groups at risk for IA is the need for life-long immunosuppression for most patients, which raises the question of optimal treatment duration and the need for secondary prophylaxis after successful treatment of an IA episode. Finally, each SOT patient category needs to be evaluated separately, because liver, lung, heart or kidney transplant recipients all have different risk factors [5, 21, 148, 149], and apparently different predispositions to the development of IA [1, 2, 5]. There is an urgent need for multicentre, international, well-orchestrated efforts on the study of IA diagnosis and management in SOT recipients to develop clear and comprehensive guidelines on the prevention, diagnosis, treatment and evaluation of therapeutic success of IA in this population.

## Data Availability

The information supporting the conclusions of this article are included within the article.

## Abbreviations

AST: Antifungal susceptibility testing
bBDG: Beta-D-glucan
CRP: C-reactive protein
CT: Computerised tomography
EORTC-MSG: European Organisation for Research and Treatment of Cancer – Mycoses Study Group
GM EIA: Galactomannan enzyme immunoassay
HCT: Hematopoietic cell transplantation
Hp: Haptoglobin
IA: Invasive aspergillosis
IMI: Invasive mold infections
ISHLT: International Society for Heart and Lung Transplantation
IV: Intravenous
L-AmB: Liposomal amphotericin B
LFD: Lateral-flow device
PAS: Periodic acid–Schiff
PCR: Polymerase chain reaction
PCT: Procalcitonin
PK/PD: Pharmacokinetic/pharmacodynamic
SOT: Solid organ transplant
TDM: Therapeutic drug monitoring
